# Letting the calcium flow

**DOI:** 10.7554/eLife.96139

**Published:** 2024-02-09

**Authors:** Régis Nouvian

**Affiliations:** 1 Institute for Neurosciences of Montpellier, Univ Montpellier, Inserm, CNRS Montpellier France

**Keywords:** hearing, hair cells, calcium channel, calcium binding protein, glutamate, Mouse

## Abstract

Two calcium-binding proteins, CaBP1 and CaBP2, cooperate to keep calcium channels in the hair cells of the inner ear open.

**Related research article** Oestreicher D, Chepurwar S, Kusch K, Rankovic V, Jung S, Strenzke N, Pangrsic T. 2024. CaBP1 and 2 enable sustained Ca_V_1.3 calcium currents and synaptic transmission in inner hair cells. *eLife* **13**:RP93646. doi: 10.7554/eLife.93646.

Hearing the voice of your lover, the symphonies of Beethoven or a fire alarm, all rely on acoustic information being translated into messages that can be understood by the brain. This process depends on sensory cells known as hair cells which are housed in a hollow within the inner ear known as the cochlea. When sound waves enter the ear, their vibrations cause fluid in the cochlea to move and bend protrusions at the top of hair cells known as stereocilia (Fettiplace, 2017). This activates hair cells and triggers calcium to enter, leading to secretion of the neurotransmitter glutamate which then activates auditory nerve fibers that convey signals to the brain (Moser et al., 2020).

The calcium influx that drives glutamate release is a critical step in the process of hearing (Pangrsic et al., 2018). In most cell types, calcium enters via channels which open upon cell activation and then inactivate themselves by closing. However, the calcium channels in hair cells (known as Cav1.3) show an interesting property: they do not inactivate and remain open for as long as the hair cell is stimulated. When the Cav1.3 channel is expressed in other cell types, the calcium channel closes itself like other calcium channels (Cui et al., 2007; Yang et al., 2006), suggesting that there is something in hair cells that stops Cav1.3 from inactivating.

Calcium channels can be inactivated in a voltage-dependent or calcium-dependent manner. Previous work found that deletion of a calcium-binding protein known as CaBP2 prevented voltage-dependent inactivation (Picher et al., 2017); a mutation in the gene encoding this protein was also shown to cause human deafness (Schrauwen et al., 2012). However, calcium-dependent inactivation was mostly unaffected by the disruption of CaBP2. Moreover, deletion of another calcium-binding protein, CaBP1, did not affect calcium-mediated inactivation, nor the voltage-dependent mechanism (Yang et al., 2018). Now, in eLife, Tina Pangrsic and colleagues from the University Medical Centre Göttingen and Max Planck Institute for Multidisciplinary Science – including David Oestreicher as first author – report that CaBP1 and CaBP2 work together to prevent calcium channels from closing during hair cell stimulation (Figure 1; Oestreicher et al., 2024).

**Figure 1.**
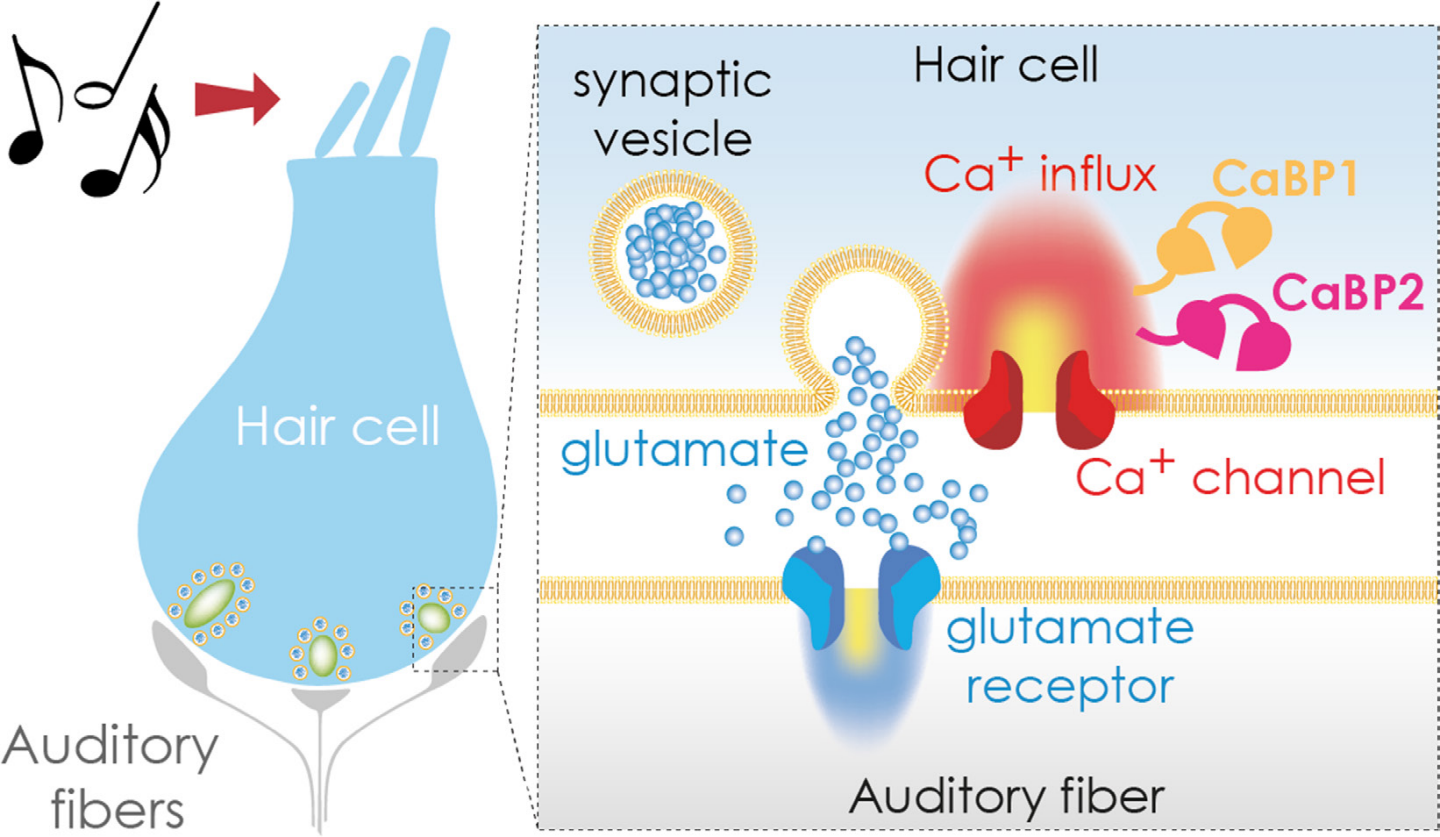
Transmitting sound from the ear to the brain. The apical region of a hair cell (left) contains bundles of stereocilia (top) that move in response to sound waves. This movement activates the hair cell, causing calcium ions to enter the cell via calcium channels (red; right hand side inset) in the basolateral region. The calcium binding proteins CaBP1 (orange) and CaBP2 (pink) both contribute to keeping the calcium channel open for as long as the hair cell is stimulated. This leads to an influx of calcium ions (Ca^2+^), which trigger the release of the neurotransmitter glutamate (blue circles), which is stored in synaptic vesicles. Once secreted, glutamate travels across a synapse (white gap) to an auditory fiber (gray) that conveys the signal to the brain, enabling the perception of sound.

The team found that deleting the genes for CaBP1 and CaBP2 simultaneously led to greater inactivation of calcium channels than deleting either gene alone. As a result of the calcium channels not staying fully open, calcium influx and glutamate release were depressed, leading to reduced activation of auditory fibers. Consequently, the neural message conveyed along the auditory pathway was degraded, resulting in severe hearing loss in the genetically modified mice. Notably, even when there was a small amount of calcium influx through the temporarily open calcium channels, glutamate secretion was still almost completely abolished. This suggests that CaBP1 and CaBP2 may affect the release of glutamate independently from their role on calcium channels.

To strengthen the data, Oestreicher et al. re-expressed the gene for CaBP2 in the mice. This led to a substantial – but not complete – rescue of calcium channel activity, glutamate secretion, auditory fiber activation and hearing. Taken together, the experiments demonstrate that both CaBP1 and CaBP2 contribute to calcium-triggered glutamate release in hair cells of the inner ear, suggesting they have partially overlapping roles.

These findings also raise exciting questions for future studies. For instance, how do CaBP1 and CaBP2 work together to control calcium channel activity? Additionally, how do these calcium-binding proteins directly influence the secretion of glutamate in addition to controlling calcium channels? Further experiments that help to answer these questions, and others, could provide more insights into how hair cells transmit sound to the brain, which could potentially help identify therapeutic targets for hearing impairments.
